# Mild-to-severe traumatic brain injury in children: altered cytokines reflect severity

**DOI:** 10.1186/s12974-022-02390-5

**Published:** 2022-02-07

**Authors:** Emer Ryan, Lynne Kelly, Catherine Stacey, Dean Huggard, Eimear Duff, Danielle McCollum, Ann Leonard, Gerard Boran, Dermot R. Doherty, Turlough Bolger, Eleanor J. Molloy

**Affiliations:** 1 Department of Paediatrics, Trinity College, The University of Dublin, Dublin, Ireland; 2 Trinity Translational Medicine Institute (TTMI), Trinity Centre for Health Sciences, Tallaght University Hospital, Dublin 24, Ireland; 3 Department of Medicine, Trinity Centre for Health Sciences, Trinity Research in Childhood Centre (TRiCC), Trinity Translational Medicine Institute, St James Hospital, Dublin 8, Ireland; 4 Paediatric Emergency Medicine, Children’s Hospital Ireland (CHI) at Tallaght, Dublin 24, Ireland; 5 Department of Biochemistry, Tallaght University Hospital, Dublin 24, Ireland; 6 Critical Care Medicine, Children’s Health Ireland (CHI) at Temple Street, Dublin 1, Ireland; 7 Department of Neonatology, CHI at Crumlin, Dublin, Ireland; 8 Department of Neonatology, Coombe Women and Infants University Hospital Dublin, Dublin, Ireland; 9 Department of Paediatric Emergency Medicine, Royal Children’s Hospital, Melbourne, Australia; 10 Department of Paediatrics, Waterford Regional Hospital, Waterford, Ireland

**Keywords:** Traumatic Brain injury, Concussion, Inflammation, Cytokines, Innate immunity

## Abstract

**Background:**

Paediatric traumatic brain injury (TBI) is recognised to have significant longer-term neurocognitive effects. Childhood is a time of high risk for head injury. Functional recovery is variable with a combination of any or all of physical, cognitive and emotional impairment. Immune activation and alteration in cytokine levels are present following TBI which may differ from adults.

**Methods:**

Pro- and anti-inflammatory cytokines including Interleukin (IL)-2, IL-4, IL-6, IL-8, IL-10, IL-17A, Tumor Necrosis Factor (TNF)-α and Interferon (IFN)-γ were examined at baseline and following in vitro treatment with endotoxin of whole blood, in the following children: severe TBI (sTBI: initial Glasgow coma scale(GCS) ≤ 8), mild TBI (mTBI; GCS 14/15) at 0-4d and at 10-14d post-TBI and compared to healthy age-matched controls.

**Results:**

The study enrolled 208 children, including 110 with TBI cohort (*n* = 104 mild; 6 severe) and controls (*n* = 98). At baseline all children with TBI had increased IL-6. The mTBI group had significantly increased IFN-γ versus controls. In sTBI at baseline, IFN-γ was decreased compared to controls. At baseline IL-8, IL-10, IL-17A, and TNF-α were decreased in mTBI compared to controls. This persisted at 2 week post-mTBI. The AUC for detecting mTBI was 0.801 CI (0.73–086) using IL6/IL10 ratio. mTBI showed a greater fold change in IL-8 and TNF-α in response to endotoxin stimulation, a response that persisted at 2 weeks. Children with sTBI did not have a significant IL-6 response to endotoxin, but did show an increase in IL-17A.

**Conclusion:**

Children with all TBI including mTBI show altered cytokine profiles and altered endotoxin responses. Although cytokines increased in sTBI especially in response to endotoxin, suppressed responses were found in mTBI coupled with persistent immune dysfunction post-injury.

**Supplementary Information:**

The online version contains supplementary material available at 10.1186/s12974-022-02390-5.

## Background

Traumatic brain injury is a global cause of preventable death and disability and a common presentation to the emergency department. Each year 3% of all children under 5 years will present to the emergency department with head injury [1]. In England and Wales, of 1.4 million emergency department visits a year for head injuries, half are under 15 years. Mild traumatic brain injury (mTBI) accounts for the majority (90%) of traumatic brain injury [2]. The paediatric brain responds differently to injury for a variety of physical and developmental reasons, as well as the maturity of the immune system [3]. Inflammatory responses to injury changes across the lifespan. Exploring the age-related inflammatory component of TBI is important for our understanding and therapeutic interventions [4]. The consequences of more severe TBI in children are catastrophic, particularly, before neuronal networks are laid down. The consequence of mTBI in children, longer term is less well-established. Despite this, there are no therapeutic agents.

Neuronal injury results in a cascade of neuroinflammation with activation of glia, the release of inflammatory mediators and the recruitment of systemic immune cells. The composition of immune cells in the peripheral blood changes as children mature, with a lymphocyte increase from 6 months to adult and a decrease in neutrophils in teenage years [5]. The immune response to infective and inflammatory stimuli changes over a lifetime. An experimental animal model shows that TBI induces widespread innate and adaptive immune changes in myeloid progenitors, thymus and T cells months after injury [6]. Following brain injury in the neonatal period, by school age, children had chronically altered cytokine profiles with elevated serum IL-2, IL-6 and IL-8 [7]. Deficiency or overexpression of IL-6 impair the recovery phase of TBI. In the acute stage of severe adult traumatic brain injury (sTBI), IL-6 and IL-8 show an early increase [8]. Cerebrospinal fluid showed high levels of IL-6 and IL-10 in earlier sTBI studies in children [9]. Serum IL-6, IL-8 and IL-10 predict poorer neurological outcomes in paediatric sTBI [10] Increased serum IL-6 in mTBI was significantly associated with the duration of symptoms in post concussive syndrome for mTBI in high school and college football players [11]. Early immune changes in the wake of TBI plays a role in the high rate of nosocomial infection in children [12].

Both IFN-γ and IL-4 have been studied in the context of TBI, but no study has generated conclusive evidence in their roles in TBI [13]. Interleukin-10 is known for its anti-inflammatory properties. In IL-10 knockout mice brain injury lesions are larger than wild-type mice [14] and increased IL-10 levels also correlate with hospital mortality in adults following sTBI [15]. A major role in inflammation is also played by TNF-α [16, 17]. In a rat model, the TNF-α antagonist Etanercept, administered early after TBI, reduced the secondary insults from oxidative stress and apoptosis with less neuronal loss [18]. The adaptive immune system, however, is less well-described in TBI. Interleukin-17A produced mainly by specialised T cells, Th17 and γδ T cells, plays a role in acute brain ischaemia, stroke [19] and multiple sclerosis [20]. It is also implicated in brain-gut signalling [21].

Here we describe the cytokine response in children with TBI compared to controls. As TBI has immunomodulating effects, we aimed to examine the cytokine profile following an additional stimulus of endotoxin in the serum of children with TBI compared to healthy controls at the time of injury. We repeated this at two weeks from injury, at timepoint when children with mTBI often appear to have recovered. Evoking and measuring an immune response in children who have sustained head injury may have a role in diagnostics, particularly in delineating recovery. This was explored using ratios of pro and anti-inflammatory markers.

## Methods

### Study population

This study was approved by the Ethics Committees of Children’s Health Ireland (CHI) at Tallaght (Ref: 2016-03 (21); approved 28.03.16) and CHI at Temple Street Dublin (Ref: 16.019; approved 23.03.16), Ireland. All families and participants received verbal and documented information on the study. Written consent was obtained prior to recruitment. The following children were enrolled: a) mTBI: Emergency department attendees with Glasgow Coma Scale 14/15; b) sTBI: admitted to the Intensive Care with a Glasgow Coma Scale ≤ 8 c) Paediatric Controls: children attending for phlebotomy or day case procedures with normal results and clinical outcomes. Children in all groups were excluded if they had recent fever or evidence of infection. Demographics that may have affect symptoms of concussion were collected included learning disability, prior medical history, visual disorders, motion sickness, migraine history, prior traumatic brain injury, family history of concussion, migraine or mental health disorders in first degree relatives (Additional file 1: Table S1). Mechanism of injury (Table 1), symptoms on presentation, at one hour and at the time of injury were recorded. A Child Sports Concussion Assessment Tool (Child-SCAT5), was recorded in children 5–12 years [22] and in children 13 years and older the SCAT5 was used [23] at the time of presentation. A post-concussive symptom inventory (PCSI) validated in children [24] was performed at the two-week assessment. Median time from injury at blood draw was four hours with an interquartile range of (1.75–22 h).

**Table 1:** Cytokines in children with TBI versus controls

Cytokine	Controls	Mild TBI	sTBI	p value*	p value **
IL-2	0.06 (0.75–2.80)	0.06 (0.86–1.34)	0.87 (0.87–0.87)	0.98	0.47
IL-4	0.80 (1.74–2.34)	0.69 (1.68–2.06)	1.72 (1.72–1.72)	0.34	0.66
IL-6	2.74 (2.19–3.28)	6.58 (4.71–8.45)	57.51 (19.32–95.70)	**0.005**	** < 0.0001**
IL-8	70.69 (61.19–80.18)	26.9 (21.02–32.97)	102.2 (3.70–200.8)	** < 0.0001**	0.38
IL-10	4.65 (3.72–5.58)	3.32 (1.59–5.05)	3.75 (1.09–6.41)	** < 0.0001**	0.72
IL-17A	14.98 (8.02–21.93)	1.48 (1.14–1.82)	1.39 (0.78–1.99)	** < 0.0001**	0.42
TNF-α	15.73 (13.51–17.95)	6.65 (5.14–8.15)	15.29 (2.33–28.24)	** < 0.0001**	0.82
IFN-γ	107.9 (75.63–140.1)	203.2 (-49.95–456.4)	17.55 (17.55–17.55)	** < 0.0001**	**0.01**

The mean, with upper and lower 95% Confidence Interval of the mean and p-values of cytokine levels (pg/ml) in plasma of children with mild Traumatic brain injury (n = 110) and severe traumatic brain injury (n = 6) versus controls (n = 98). Cytokine values in bold denote p < 0.05. *p value: comparing controls to mTBI; ** p value: comparing sTBI to mTBI

### Sample preparation

Blood samples (3 mL) for in vitro experiments were collected in a sodium citrate anti-coagulated blood tube and processed within two hours of phlebotomy. Whole blood was incubated at 37 °C for 1 h untreated (vehicle) or with Lipopolysaccharide (LPS; *E.coli* 0111:B4: SIGMA Life Science, Wicklow, Ireland) 10 ng/ml. After incubation the samples were centrifuged at 10,000 rpm for 10 min at room temperature. The plasma supernatant was stored at − 80 °C for subsequent batch analysis [25].

### Cytokine analysis

The following cytokines were evaluated using the MSD®MULTI-SPOT assay system from MesoScale Discovery, Rockville, MD, USA (www.meso-scale.com): IL-2, IL-4, IL-6, IL-8, IL-10, IL-17A, IFN-γ TNF-α [25]. Peripheral blood plasma was transferred to a 96 well MSD plate and these cytokines were assessed as per manufacturer's instructions. Assays were transferred to five U-PLEX platform plates with calibration curves showing expected signals, sensitivity precision, and accuracy. Sensitivities were < 1 pg/ml for many assays. All assays used the same diluents, diluting linearly from 1–fourfold. Non-specific binding between assays was typically < 0.1%. The assay reproducibility was calculated using the intra variation of the standard curves was shown to be within an acceptable range. The median of the lower limit of detection for all 5 plate runs were as follows: IL-2, IL-4, IL-6, IL-8, IL-10, IL-17A, IFN-γ, TNF-α, 0.87, 1.72, 0.84, 0.38, 0.62, 1.15, 17.55, 2.39 respectively, with a closely aligned average value, demonstrating the reproducibility of the assay. Where the sample was below the lower limit of detection the value of the lower limit of detection for that assay was substituted [26]. No samples reached an upper limit of detection.

### Statistics

Descriptive statistics were used to describe the demographics of the cohort. The Kolmogorov–Smirnov test was used to check normality. Statistical analysis was performed using Mann Whitney tests to compare mean ranks between two independent cohorts. Significance was defined as p = 0.05. Results shown are expressed as mean ± standard error of the mean (SEM) unless otherwise stated. Receiver operator curves were generated from baseline unstimulated serum cytokine levels. Data were analysed with GraphPad Prism v.7.

## Results

### Clinical parameters

There were 238 samples collected from 208 children enrolled in this study, including 110 children with TBI (mTBI n = 104; sTBI *n* = 6) and 98 paediatric controls. In the mTBI group, 12 recruited patients declined phlebotomy and 30 mTBI children had repeat serology at 2 weeks. The mean (SD) age of children enrolled was as follows: sTBI (7.9 ± 5.0)y, male (*n* = 5), mild TBI (11.2 ± 4.0)y male (*n* = 69) and controls (8.0 ± 4.2)y, male (*n* = 54). The sTBI cohort had a Glasgow Coma Scale (GCS) of less than 8 and required intubation, ventilation and an Intensive Care management. The causes of sTBI included falls from windows, car accidents, a crush injury and one fall. All had intracranial bleeding on CT imaging. Children in the mild TBI group had a GCS of 14/15 with the following causes: sports (*n* = 53; 51%), accidents including falls (*n* = 44; 42.3%) and road traffic accidents (*n* = 7;6.7%). A loss of consciousness was described in 16.6% and 35.6% had amnesia. In the mild TBI group 26 (28.8%) underwent CT imaging and 4 were abnormal: depressed skull fracture and intracranial bleed; small left frontotemporal contusion/contra-coup haemorrhagic lesion and non-displaced skull fractures (*n* = 2). In the mTBI cohort vomiting occurred in 41.8% with more than 3 episodes in 27.8%. Full blood counts were available for 77 children at the same blood draw; median (interquartile range) white cell counts 8.4 × 10^9^/L (6.5- 11 × 10^9^/L), neutrophils 4.5 × 10^9^/L (3.3–8.3 × 10^9^/L), lymphocytes 2.1 × 10^9^/L (1.5–2.6 × 10^9^/L.).

### Cytokines responses in Children with Traumatic brain injury versus controls at baseline

**Fig. 1:**
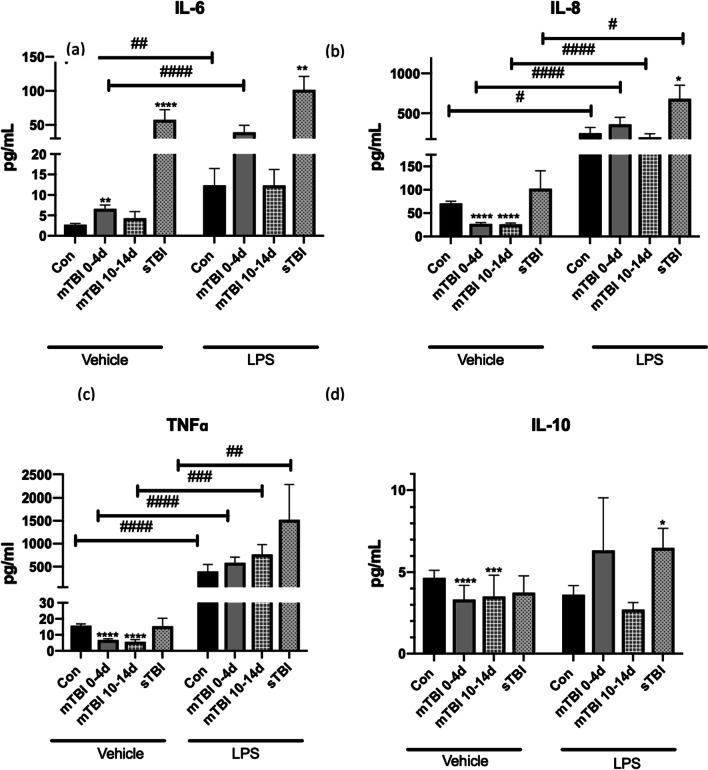
Cytokines: **a** Interleukin (IL)-6, **b** IL-8, **c** TNFα and **d** IL-10 responses in Paediatric Traumatic brain injury. Serum of children with mild TBI (mTBI) at the time of presentation and at 2 weeks as well as children with severe TBI (sTBI) at baseline and in response to LPS. Values expressed as pg/ml. Samples size (Vehicle: *n* = 104 controls, *n* = 98 mTBI at 0-4d, n = 29 mTBI at 10-14d, *n* = 6 severe TBI. LPS: *n* = 11 controls, *n* = 18 mTBI at 0-4d, *n* = 9 mTBI at 10-14d, n = 4 severe). *p < 0.05, **p < 0.01, ***p < 0.001, ****p < 0.0001, patient cohort compared to controls. # = p < 0.05 response to LPS from baseline. **a** **p = 0.005 mTBI 0-4d compared to controls, ****p < 0.0001 control compared to sTBI at baseline, ##*p* = 0.001 controls pre and post LPS stimulation. #### p < 0.0001 mTBI 0-4d pre and post LPS. **p = 0.0015 controls compared to sTBI within LPS group. **b** ****p < 0.0001 mTBI 0 = 4d compared to controls, ****p < 0.0001 mTBI at 10-14d compared to controls, *p = 0.035 controls compared to sTBI in response to LPS. ^#^*p* = 0.02 controls pre and post LPS, ^#^*p* = 0.02 sTBI pre and post LPS ^####^*p* < 0.0001 mTBI pre and post LPS ^####^*p* < 0.0001 mTBI at 10-14d pre and post LPS. **c** ****p < 0.0001 mTBI 0 = 4d compared to controls, ****p < 0.0001 mTBI at 10-14d compared to controls ^##^*p* = 0.009 sTBI pre and post LPS ^###^*p* = 0.0001 mTBI at 10-14d pre and post LPS ^####^*p* < 0.0001 mTBI pre and post LPS, LPS ^####^*p* < 0.0001 controls pre and post LPS. **d** ****p < 0.0001 mTBI 0-4d compared to controls, ***p = 0.0004 mTBI at 10-14d compared to controls, *p = 0.037 controls compared to sTBI within LPS group

**Fig. 2:**
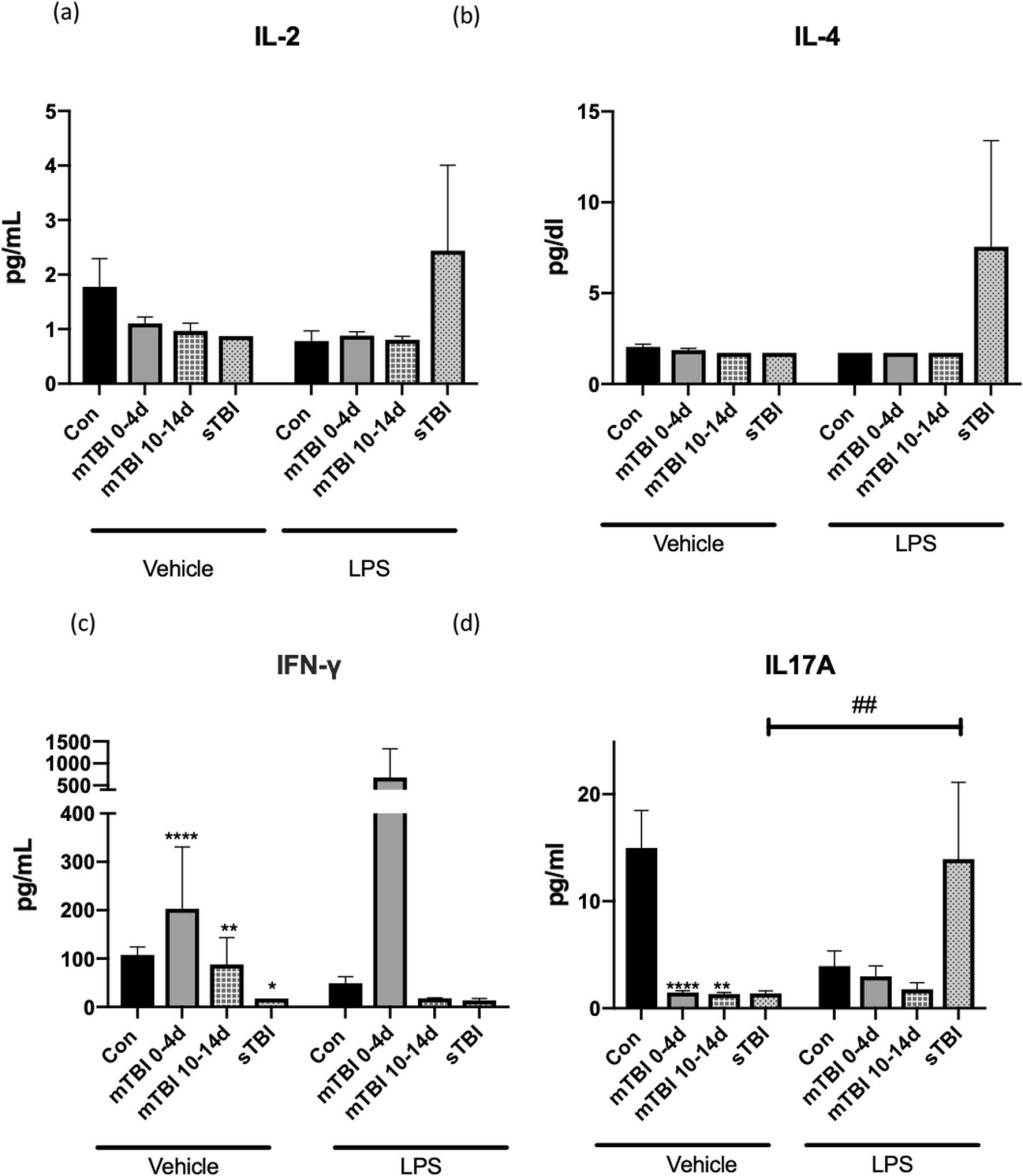
Cytokines: **a** Interleukin 2 (IL)-2, **b** IL-4, **c** IFNγ and **d** IL-17A responses in Paediatric Traumatic brain injury*.* Serum of children with mild TBI (mTBI) at the time of presentation and at 2 weeks as well as children with severe TBI (sTBI) at baseline and, in response to LPS. Values were expressed as pg/ml. Samples size (Vehicle: *n* = 104 controls, *n* = 98 mTBI at 0-4d, *n* = 29 mTBI at 10-14d, *n* = 6 severe TBI. LPS: *n* = 11 controls, *n* = 18 mTBI at 0-4d, *n* = 9 mTBI at 10-14d, *n* = 4 severe). *p ≤ 0.05*, **p < 0.01, ***p < 0.001, ****p < 0.0001, patient cohort compared to controls. # = p < 0.05 response to LPS from baseline. **a**, **b** there was no statistical difference between groups. **c** ****p < 0.0001 mTBI at baseline compared to controls, **p = 0.0053 mTBI at 10-14d compared to controls, *p = 0.01 severe compared to controls **d** **p = 0.0097 control compared to mTBI at 10-14d, ****p < 0.0001 control compared to mTBI at 0-4d, ^##^*p* = 0.0048 sTBI pre and post LPS stimulation

Interleukin-6 was raised in all children with TBI compared to controls and was significantly higher in sTBI versus mTBI (p < 0.0001; Fig. 1). All children with sTBI had IL-6 levels of > 20 pg/ml and no children with mTBI had values over 45 pg/ml. This was highest in the first 0–12 h after injury (Additional file 2: Fig. S1) and was not significantly raised after this time point. At a cut-off value of 20 pg/ml IL-6 was 100% sensitive and 99% specific for sTBI and 91% sensitive for mTBI detection but non-specific. Interferon-γ was decreased in sTBI (p = 0.01) but raised in mTBI (p < 0.0001; Table 1, Fig. 2). Although in children with sTBI IL-8, IL-10 IL-17A and TNF-α, did not change these cytokines were decreased in children with mTBI versus controls (Table 2 & Figs. 1, 2). There were no significant differences seen in IL-2 or IL-4 in mild or sTBI compared to controls (Table 2 & Fig. 2). While patients with mTBI had significant but less potent increases in IL-6 concentrations than sTBI, they had a rise in IFN-γ, and reduction in IL-8, IL-10, IL-17A and TNF-α concentrations. There was no significant difference between gender (Additional file 2: Fig. S2). There were higher levels of IL-8 and TNF-α in younger boys (Additional file 2: Fig. S3) while IL-10 was lower in older girls (Additional file 2: Fig. S4).

**Table 2:** Receiver operator characteristic curves for cytokines of children with mild Traumatic brain injury and controls

Cytokine	mTBI at 0-4d AUC	mTBI at 10-14d AUC
Prediction of mTBI		
IL-6	0.61	0.464
TNF-α	0.204	0.163
IL6/IL8	0.829	0.744
IL6/IL10	0.801	0.686
IL6/IL-17A	0.744	0.621
IL6/IL10/IL-17A	0.832	0.746

### Cytokines to identify traumatic brain injury in children at presentation

To further examine the utility of cytokine measurement and ratios of pro- and anti-inflammatory markers in the setting of TBI, receiver operating characteristic curves were used to select patients with TBI compared to controls (Table 2, Additional file 2: Fig. S5). Severe TBI were distinguished from control patients by IL-6 with 100% sensitivity and specificity, with an Area Under the Curve (AUC) of 1. Mild TBI is less obvious clinically and biochemically and the presence of mild TBI was indicated by IL-6 with an AUC of 0.61 CI (0.54–0.691). The ratio of IL-6/IL-8 gave the highest AUC of 0.829 to select mild TBI. Since IL-10 and IL-17A were decreased in both mild and severe TBI compared to controls we examined the utility of using these to create a ratio which would be useful across the continuum from mild to severe. Using IL-6/IL-10 increased the AUC to predict mTBI to 0.801 CI (0.738–0864). Using IL-6/IL-10/IL-17A the AUC was 0.832 at 0-4d.

### Effects of ex-vivo LPS stimulation on Cytokine responses in children with TBI and controls

To challenge the immune system in the setting of TBI whole blood was stimulated with LPS. In sTBI, LPS stimulation caused a significant rise in IL-8, TNF-α and IL-17A from baseline (*p* < 0.000001, *p* < 0.000001, *p* = 0.0048). Baseline levels of IL-6 in the sTBI cohort were high compared to both mTBI and controls and were not significantly increased with LPS (Fig. 1). Prior to stimulation with LPS, IL-8 and IL-10 levels were not significantly higher in sTBI compared to controls (*p* = 0.38, *p* = 0.7). Following LPS stimulation, while all groups had increased cytokine production there was a significant increase in IL-8 and IL-10 levels in sTBI compared to stimulated control samples (*p* = 0.035, *p* = 0.037). In paediatric controls and in children with mTBI, LPS stimulation significantly increased IL-8, TNF-α and also IL-6 levels (< 0.0001, < 0.0001, < 0.0001; Fig. 1). In children with mTBI baseline IL-8 and TNF-α levels were low but were significantly responsive to LPS beyond the response seen for controls (Table 2). A significant response to LPS stimulation for IL-8 and TNF-α (*p* < 0.0001, *p* = 0.0002; Table 2) was seen in the serum taken from children with mTBI at 10–14 days from injury. Interleukin-2 and IL-4 did not respond to LPS stimulation in any group. Stimulation with LPS unmasked a hyperresponsiveness of the immune system in children who had both sTBI and mTBI. Children with mTBI specifically had lower levels of cytokines until stimulated with LPS.

### Persistent inflammatory response

Due to the persistence of the immune response for an undefined period of time after TBI, we followed the immune response in these patients for two weeks. In children with mTBI at 2 weeks, IL-17A, IL-8, IL-10 and TNF-α levels remained significantly decreased (*p* = 0.009, *p* < 0.0001, *p* = 0.0006, *p* < 0.0001, respectively), while IL-6 returned to baseline (Figs. 1 and 2). In mTBI, IL-8 responsiveness to LPS peaked at 0–4 days but remained above control levels at 10–14 days (Additional file 3: Table S2). In mTBI LPS induced a threefold increase in IFN-γ levels on 0–4 day. This phenomen was not seen at 2 weeks where LPS induced a decreased response. There was a greater TNF-α production in response to LPS in mTBI at 10–14 days than at 0–4 day and versus controls (Table 2). At 10–14 days from injury the serum of children with mild TBI showed persistent cytokine levels with an ROC curve using a ratio of IL-6/IL-10/IL-17A to give an AUC of 0.746. The cytokine production seen in children with mTBI persisted for 2 weeks as well as the response to LPS.

## Discussion

At the time of injury, in children with both sTBI and mTBI, IL-6 was significantly elevated compared to controls. When stimulated ex-vivo with LPS, in sTBI and not mTBI, significant elevations of IL-8 and IL-10 were seen compared to controls. It is well-known that there is an alteration in the immune profile following sTBI in adults and children [12]. The injury profile in mTBI is not on a continuum with sTBI, mTBI having no gross injuries on imaging. In recent studies, blast injury has too, been shown to be distinct [27]. An adult mouse model has shown increased remote lung neutrophil migration in the cohort with mTBI compared to controls [28]. No pediatric study has shown sustained systemic immune change following mTBI. This mTBI cohort showed significantly lower IL-8, IL-10 IL-17A and TNF-α at the time of injury. However, when stimulated with LPS, these children with mTBI had greater IL-8 and TNF-α responses, both in serum from the time of injury and serum at 2 weeks, showing persistent immune activation, at a time when the majority of children show clinical recovery. Sex hormones have a role to play in TBI, with the oestrogen in female puberty being protective [29]. While there were no significant differences in gender, older boys had lower IL-8 and TNF-α responses, while older females had lower IL-10. Due to the smaller number of female patients in the study, and due to the heterogeneous nature of injuries in the cohort this should be interpreted with caution. Serum taken 2 weeks from injury showed the same pattern of significantly decreased IL-17A, IL-8, IL-10 and TNF-α levels, while IL-6 had returned to baseline. The systemic immune response to mTBI in children is a reduction of cytokine production by myeloid cells. While this effect persists, if provoked by endotoxin, the response of the peripheral myeloid cells in children with mTBI is exaggerated.

There are gaps in our understanding in how to modify the innate immune response to trauma to better the outcomes in TBI across the spectrum, particularly in children where the innate immune responses are different. Limiting the pro-inflammatory response and promoting anti-inflammatory properties is key to therapeutics. Defining these responses and timelines are important for secondary injury, particularly in the milder TBI children, not previously known to have persistent immune dysregulation. Early measurement of innate immune function may better help to risk stratify in mTBI.

Interleukin-6 was elevated in all children with TBI and predicted sTBI. IL-6/IL-10 ratio enhanced the prediction of mTBI relative to IL-6 alone. In sports related concussion IL-6 levels correlated with symptom burden in young adults [11]. While IL-6 is elevated in mTBI in adult studies, it has not correlated with cognitive impairment [30], a common component of post concussive syndrome. Gill and colleagues showed significant increases in IL-6, IL-10 and TNF-α during blast exposure in military training [31]. In one study IL-6 and TNF-α were raised in mTBI, but TNF-α was only raised in those with evidence of injury seen on CT, and not in those with injury only evident on MRI [32]. In our study IL-6 elevations did not persist at 2 weeks. Interestingly exercise prior to injury in animal models have been shown to attenuate levels of IL-6 in response to mTBI [33], and also the effect of the injury [34].

In childhood, sTBI is associated with increased serum IL-8 and predicted a worse outcome [10]. In neonatal brain injury elevated IL-8 and IL-10 correlated with poor outcome and severity of injury [35]. Most TBI studies of cytokine responses have been in sTBI but not mTBI, where there is no overt intracranial bleeding, and the mechanism of injury is different. We demonstrated an opposite effect in this cohort with significantly lower levels of IL-8 and IL-10 in our pediatric mTBI cohort at baseline when compared to controls. An adult study looking at blast-related TBI in the military found similar responses to our study: IL-8 decreased from pre-deployment levels in those who suffered blast injury [27]. Interestingly this effect was more profound in those younger than 25 years. This may be an effect of the injury type or of age or may also suggest the response to all brain injury is not on a continuum.

There was a significant and sustained decrease in IL-17A following TBI. Only in sTBI did LPS mediate an IL-17A response to stimulation. Interleukin-17A is associated with TBI severity and also progression in multiple sclerosis. It has a role in mediating the blood–brain barrier [36]. A sTBI model in rats showed increased IL-17 production [37]. The role of the adaptive immune system in TBI, particularly in repeated mTBI needs further clarification.

Two weeks from injury there was a sustained decrease in IL-8, IL-10 IL-17A and TNF-α in the mTBI cohort. Animal studies demonstrate persistent immune dysfunction in sTBI [6] and chronic changes associated with repeated head injury [38]. The mouse model of head injury showed chronic persistent changes in thymic and T-cell function, neutrophil and monocyte function, and proinflammatory cytokine production including TNF-α, at 60 days [6]. In moderate/severe blast injury there was a reduction in IL-10, IL-17A and TNF-α even at a year from injury reflecting persistent inflammation [39]. Following neonatal encephalopathy children have persistent immune dysfunction much longer, at school-age [7]. Jeschke and colleagues demonstrated altered immune response in the three years following a severe physical burn injury in children [40]. The response of the immune system over time to one TBI and repeated insults may be amenable to immunomodulation.

Immunomodulatory effects of TBI are demonstrated in both cohorts. Provocative testing with LPS stimulation of samples from children with TBI unmasks immune dysregulation and may aid diagnostics. sTBI had significantly elevated IL-8 and IL-10 response to LPS while mTBI had a significant and sustained IL-6 and IL-8 response to LPS challenge, at time of injury and at two weeks. The greater TNF-α response at 10–14 days was specific to mTBI, suggesting priming of peripherally circulating immune cells. Prior studies of TNF-α responsive to LPS looked only at sTBI and not mTBI. A blunted TNF-α response in sTBI to LPS stimulation has been previously demonstrated in sepsis and trauma in children [41, 42]. This phenomenon results in increased nosocomial infections [43]. Our study is the first to look at children with mTBI/concussion which shows priming of the TNF-α response from myeloid cells to LPS at two weeks, with an opposite and greater response to LPS challenge, at time when the majority of children appear to have recovered.

Innate immune function in children is distinctly different to adults. Ageing is associated with low grade chronic systemic inflammation with elevated IL-6 and C-reactive protein [44]. Studying the effects of TBI in children naïve to chronic disease offers a unique opportunity to look at TBI in isolation. Understanding the pathophysiological and post inflammatory changes associated with paediatric TBI may facilitate the understanding of potential new drug therapies in the initial and chronic phases of TBI. Even the use and timing of basic anti-inflammatory medication is poorly understood. This study was limited by the small number of sTBI, but the large number in the mTBI cohort was a strength in this under studied population.

## Conclusion

This study demonstrated immune dysfunction not only in sTBI but also in mTBI. It established that immune dysfunction is persistent following paediatric mTBI. Interleukin-6 was elevated across the spectrum of TBI. The effect of TBI on systemic inflammation warrants serial follow up for months and years. Evidence of injury in mTBI is occult, usually described subjectively by symptom report. A strong biomarker of mTBI has alluded scientific research, however provocative immune testing may be most beneficial. Further validation of these cytokines is key to understanding the mechanisms that are also a target of immunomodulatory therapy.

## Supplementary Information

**Additional file 1. Table S1.** Detailed demographics of children with TBI.

**Additional file 2. Figure S1. **Cytokines in Paediatric Traumatic brain injury at time epochs from injury. **Figure S2.** Cytokines in Paediatric Traumatic brain injury by gender. **Figure S3.** Cytokines in Male Paediatric Traumatic brain injury by age group. **Figure S4.** Cytokines in Female Paediatric Traumatic brain injury by age group. **Figure S5.** Receiver operator characteristic curves for predicting presence **a**, **b** and absence **c**, **d** of mTBI compared to controls.

**Additional file 3. Table S2. **LPS induced cytokine responses in children with mTBI versus controls.

## Data Availability

The experimental data used to support the findings of this study are available from the corresponding author upon request.

## Abbreviations

CT: Computerised tomography
GCS: Glasgow coma scale
IFN-γ: Interferon-γ
IL: Interleukin
LPS: Lipopolysaccharide
mTBI: Mild traumatic brain injury
PCSI: Post concussive symptom inventory
SCAT: Sports concussion assessment tool
sTBI: Severe traumatic brain injury
TBI: Traumatic brain injury
TNF-α: Tumour necrosis factor-α
