# Supplementary material for: Mild-to-severe traumatic brain injury in children: altered cytokines reflect severity
Source: J Neuroinflammation. 2022 Feb 7;19:36. doi: 10.1186/s12974-022-02390-5 (PMC8822689; doi:10.1186/s12974-022-02390-5)
Supplement: Supplementary file 1 — Additional file 1. Table S1. Detailed demographics of children with TBI. [file 12974_2022_2390_MOESM1_ESM.docx]

**Additional file 1: Table S1: Detailed demographics of children with TBI.**

| Special Care/Neonatal ICU admission | 12 | 12.0% | 0 | 0 |
| --- | --- | --- | --- | --- |
| Resus as infant | 2 | 2.0% | 1 | 16.6% |
| cerebral palsy | 1 | 1.0% | 0 | 0 |
| Learning or Developmental disorder |  |  |  |  |
| Speech Delay | 11 | 10.8% | 0 | 0 |
| Special Needs Assistant at school | 5 | 4.9% | 1 | 16.6% |
| Autism Spectrum Disorder | 3 | 2.9% | 1 | 16.6% |
| Attention Deficit Hyperactivity Disorder | 4 | 4.0% | 0 | 0 |
| Dyspraxia | 3 | 2.9% | 0 | 0 |
| Dyslexia | 11 | 10.8% | 0 | 0 |
| Any learning/developmental disability* | 39 | 25.9% | 1 | 16.6% |
| Medical conditions |  |  |  |  |
| Epilepsy Diagnosis | 1 | 1.0% | 0 | 0 |
| Previous Seizure | 5 | 4.9% | 0 | 0 |
| Previous Concussion | 27 | 27.0% | 0 | 0 |
| Depression or anxiety | 10 | 9.8% | 1 | 16.6% |
| Travel sickness | 14 | 14.7% | 0 | 0 |
| Wears glasses | 16 | 16.3% | 0 | 0 |
| Suffers from headaches migraine | 23 | 22.5% | 0 | 0 |
| Family History |  |  |  |  |
| Family hx Migraine 1^st^ degree relative | 38 | 37.6% | 2 | 33.2% |
| Family hx depression 1^st^ degree relative | 18 | 17.5% | 1 | 16.6% |
| Family hx concussion 1^st^ degree relative | 17 | 17.2% | 1 | 16.6% |

*any learning /developmental disability includes Speech delay, a special needs assistant at school, Autistic spectrum disorder, Attention Deficit Hyperactivity Disorder, dyspraxia, dyslexia.
