# Supplementary material for: Mild-to-severe traumatic brain injury in children: altered cytokines reflect severity
Source: J Neuroinflammation. 2022 Feb 7;19:36. doi: 10.1186/s12974-022-02390-5 (PMC8822689; doi:10.1186/s12974-022-02390-5)

**Additional file 2: Figure S1: Cytokines in Paediatric Traumatic brain injury at time epochs from injury.** Values expressed as pg/ml. Samples size (n= 98 controls, n= 53 mTBI at 0-12hr, n= 15 mTBI at 12-48hr, n= 21 mTBI at 48-96hr, *p <0.05, **p<0.01, ***p<0.001, comparison with control sample. Interleukin (IL)-10 p *** <0.0001 mTBI 0-12hr, 12-48hr and 48-96hr. Interferon 𝛄 ***p = 0.0003 mTBI 0-12hr, p=0.09 12-48hr, **p = 0.0059 48-96hr. IL-17𝛂 **p = 0.0056 mTBI 0-12hr, p = 0.21 12-48hr, *p = 0.0143 48-96hr. IL-4 p = 0.227 mTBI 0-12hr, p = 0.101 12-48hr, p = 0.775 48-96hr. IL-6 ***p <0.0001 mTBI 0-12hr, p <0.848 12-48hr, p = 0.42 48-96hr. IL-8 ***p <0.0001 mTBI 0-12hr, 12-48hr and 48-96hr . Tumour Necrosis Factor 𝛂 *** p <0.0001 mTBI 0-12hr, 12-48hr and 48-96hr


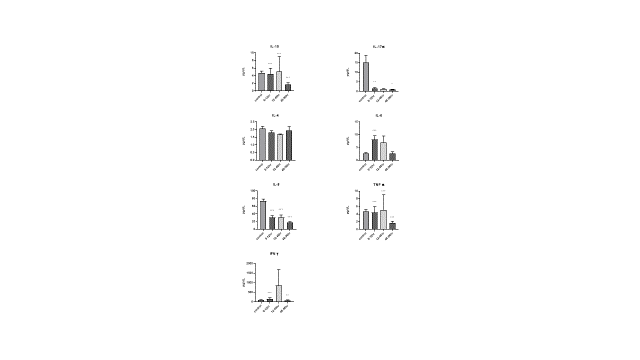


**Additional file 2: Figure S2: Cytokines in Paediatric Traumatic brain injury by gender.** Values expressed as pg/ml. Samples size (n= 32 female, n= 58 male) No significant results.

Interleukin (IL)-10 p = 0.542 Interferon 𝛄 p = 0.336. IL-17𝛂 p = 0.287. IL-4 p = 0.6. IL-6 p = 0.938. IL-8 p = 0.322. Tumour Necrosis Factor 𝛂 p = 0.843


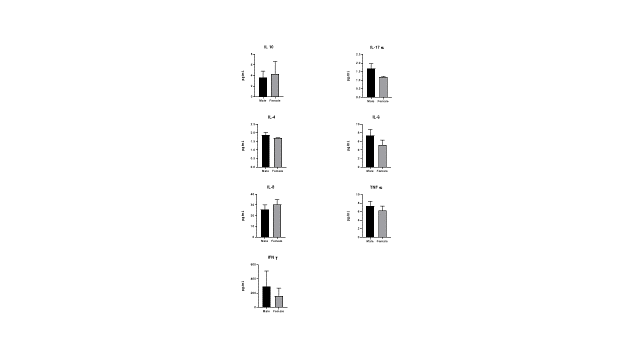


**Additional file 2: Figure S3: Cytokines in Male Paediatric Traumatic brain injury by age group.** Values expressed as pg/ml. Samples size (n= 14 0-10yrs, n= 44 over 10yrs), *p <0.05, **p<0.01, ***p<0.001, ****p <0.0001.

Interleukin (IL)-10. P = 0.2 Interferon 𝛄 p = 0.758. IL-17𝛂 p > 0.9999. IL-4 p = 0.1863. IL-6 p = 0.938. IL-8 * p = 0.039. Tumour Necrosis Factor 𝛂 * p = 0.018


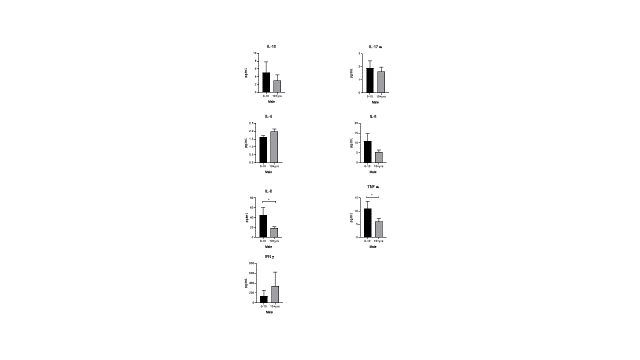


**Additional file 2: Figure S4: Cytokines in Female Paediatric Traumatic brain injury by age group.** Values expressed as pg/ml. Samples size (n= 13 0-10yrs, n= 19 over 10yrs).

Interleukin (IL)-10 * p = 0.039. Interferon 𝛄 p = 0.771. IL-17𝛂 p > 0.9999. IL-4 p = 0.406. IL-6 p = 0.208. IL-8 p = 0.238. Tumour Necrosis Factor 𝛂 p = 0.496


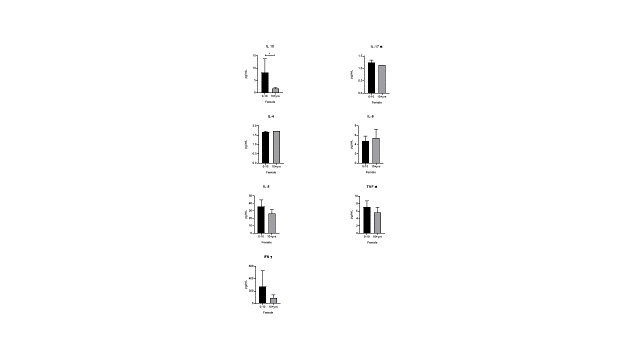


**Additional file 2: Figure S5: Receiver operator characteristic curves for predicting presence (a-b) and absence (c-d) of mTBI compared to controls***:* Receiver operator characteristic curves for predicting presence of mTBI with (a) IL-6 alone (Area under the Curve: 0.61), and (b) IL6 as a ratio divided by IL8. (Area under the Curve: 0.829)*.*

1. IL-6 (b) IL6/ IL8


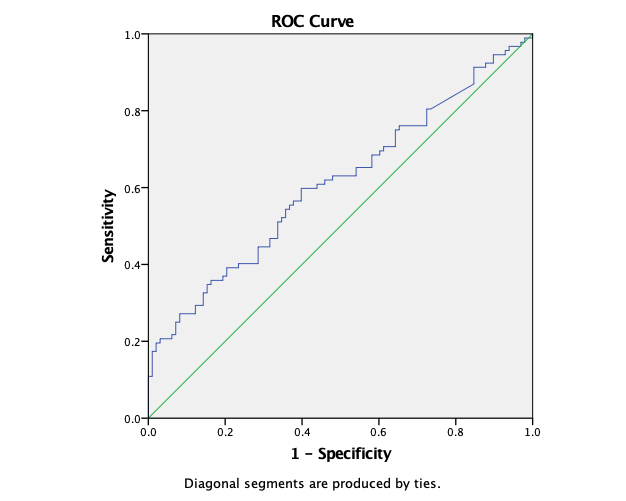

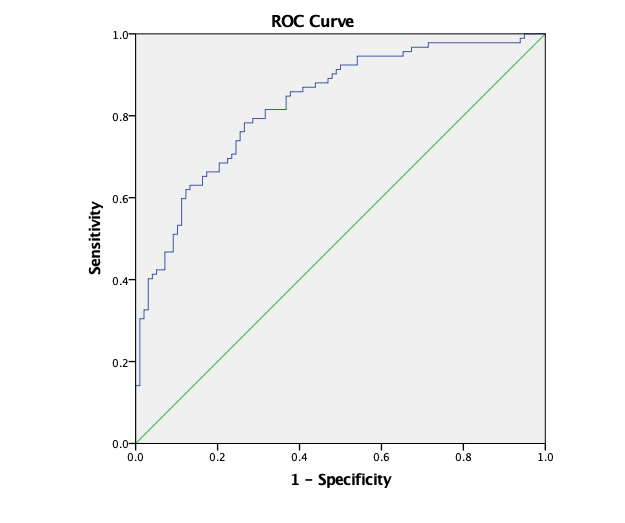

Supplement: Supplementary file 2 — Additional file 2. Figure S1. Cytokines in Paediatric Traumatic brain injury at time epochs from injury. Figure S2. Cytokines in Paediatric Traumatic brain injury by gender. Figure S3. Cytokines in Male Paediatric Traumatic brain injury by age group. Figure S4. Cytokines in Female Paediatric Traumatic brain injury by age group. Figure S5. Receiver operator characteristic curves for predicting presence a, b and absence c, d of mTBI compared to controls. [file 12974_2022_2390_MOESM2_ESM.docx]
