# Supplementary material for: Mild-to-severe traumatic brain injury in children: altered cytokines reflect severity
Source: J Neuroinflammation. 2022 Feb 7;19:36. doi: 10.1186/s12974-022-02390-5 (PMC8822689; doi:10.1186/s12974-022-02390-5)
Supplement: Supplementary file 3 — Additional file 3. Table S2. LPS induced cytokine responses in children with mTBI versus controls. [file 12974_2022_2390_MOESM3_ESM.docx]

**Additional file 3: Table S2: LPS induced cytokine responses in children with mTBI versus controls**

| **IL-8** | **Baseline** | **LPS** | **Fold Change** | **p value** |
| --- | --- | --- | --- | --- |
| **Control** | 70.69 (61.19 – 80.18) | 251.18 (93 – 409.4) | 3.55 | - |
| **mTBI 0-4d** | 26.99 (21.02 – 32.97) | 361.52 (178.9 – 544.2) | 13.39 | - |
| **mTBI 10-14d** | 25.935 (19.97 – 31.90) | 198.11 (92.33 – 303.9) | 7.63 | <0.0001 |
|  |  |  |  |  |
| **TNF-α** | **Baseline** | **LPS** | **Fold Change** |  |
| **Control** | 15.73 (13.51 – 17.95) | 393.26 (49.06 – 737.5) | 25 | - |
| **mTBI 0-4d** | 6.647 (5.14 – 8.15) | 580.82 (322.5 – 839.1) | 87.47 | - |
| **mTBI 10-14d** | 5.68 (2.93- 8.43) | 764.665 (267.7 – 1262) | 134.62 | 0.0002 |

The mean of cohort IL-8 and TNF-α levels (pg/ml) in plasma of children with mild Traumatic brain injury at baseline and at two weeks and the control group, before and after LPS stimulation with the fold change in mean. Interleukin 8 (IL-8), Tumour necrosis factor α (TNF-α). Chi Squared test, p > 0.05
